# What drives different treatment choices? Investigation of hospital ownership, system membership and competition

**DOI:** 10.1186/s13561-021-00305-3

**Published:** 2021-02-16

**Authors:** Esra Eren Bayindir, Jonas Schreyögg

**Affiliations:** Hamburg Center for Health Economics, University of Hamburg, Hamburg, Germany

**Keywords:** Hospital ownership type, System membership, Competition, Treatment choices

## Abstract

**Background:**

Differences in ownership types have attracted considerable interest because of policy implications. Moreover, competition in hospital markets is promoted to reduce health care spending. However, the effects of system membership and competition on treatment choices of hospitals have not been considered in studying hospital ownership types. We examine the treatment choices of hospitals considering ownership types (not-for-profit, for-profit, and government), system membership, patient insurance status (insured, and uninsured) and hospital competition in the United States.

**Methods:**

We estimate the probability of according the procedure as the treatment employing logistic regression. We consider all procedures accorded at hospitals, controlling for procedure type and diagnosis as well as relevant patient and hospital characteristics. Competition faced by hospitals is measured using a distance-weighted approach separately for procedural groups. Patient records are obtained from State Inpatient Databases for 11 states and hospital characteristics come from American Hospital Association Annual Survey.

**Results:**

Not-for-profit hospitals facing low for-profit competition that are nonmembers of hospital systems, act like government hospitals, whereas not-for-profits facing high for-profit competition and system member not-for-profits facing low for-profit competition are not statistically significantly different from their for-profit counterparts in terms of treatment choices. Uninsured patients are on average 7% less likely to be accorded the procedure as the treatment at system member not-for-profit hospitals facing high for-profit competition than insured patients. System member not-for-profit hospitals, which account for over half of the observations in the analysis, are on average 16% more likely to accord the procedure as the treatment when facing high for-profit competition than low-for-profit competition.

**Conclusions:**

We show that treatment choices of hospitals differ by system membership and the level of for-profit competition faced by the hospitals in addition to hospital ownership type and health insurance status of patients. Our results support that hospital system member not-for-profits and not-for-profits facing high for-profit competition are for-profits in disguise. Therefore, system membership is an important characteristic to consider in addition to market competitiveness when tax exemption of not-for-profits are revisited. Moreover, higher competition may lead to increasing health care costs due to more aggressive treatment choices, which should be taken into account while regulating hospital markets.

**Supplementary Information:**

The online version contains supplementary material available at 10.1186/s13561-021-00305-3.

## Background

Whether not-for-profit hospitals differ from their for-profit counterparts has been examined without reaching any conclusive results due to abundance of aspects to consider. Recently, distribution of the Coronavirus Aid, Relief, and Economic Security Act Provider Relief Fund to prevent health care providers from capsizing during the coronavirus pandemic among hospitals in the United States (US) brought the issue to public attention again. Twenty large hospital chains, many of which are not-for-profits, with more than $108 billion in cash received more than $5 billion from the first rounds of funding [1] drawing attention to whether these not-for-profits deserve the federal tax exemptions they enjoy and whether they are any different from their for-profit counterparts.

The main legal difference between not-for-profit and for-profit hospitals is that for-profits distribute accounting profits to shareholders and not-for-profits enjoy tax exemptions and may receive private donations [2]. Differences among hospitals by ownership type have been studied extensively. Newhouse [3] claims that not-for-profits maximize own output, which is defined as a weighted average of quantity and quality of care supplied by the hospital unlike for-profit hospitals, which maximize expected profits. Weisbrod [4] argues that not-for-profits maximize the output of the market in which they operate, hence they are expected to maximize the welfare of the community subject to the constraints they face [2]. On the other hand, Pauly and Redisch [5] claim that not-for-profits and for-profits both maximize their expected profits, whereas Hirth proposes that some not-for-profits do not have the objective of maximizing profits, therefore they are true not-for-profits whereas some of them are for-profits in disguise [6, 7].

Previous research on ownership of hospitals has largely focused on financial measures such as costs, profits and responsiveness to financial pressure and it has been traditionally concluded that there are few differences between not-for-profits and for-profits [8–11]. For-profit hospitals are more likely to respond to incentives compared to not-for-profit and government hospitals [12] and to upcode^1^ to generate higher profits than not-for-profits [14]. Considering service provision probabilities by hospital ownership type, for-profit hospitals are found to be the most likely to offer profitable services and the least likely to provide unprofitable services whereas not-for-profit hospitals lie in between for-profit and government hospitals in the US [15–17]. Uninsured patients are accorded fewer procedures as the treatment than insured patients controlling for health status [18]. Not-for-profit hospitals significantly differ from for-profits in terms of treatment choices of less profitable patients, and they lie between for-profit and government hospitals in terms of profit seeking behavior considering treatment choices as well [19].

Not-for-profit hospitals are expected to provide more uncompensated care than their for-profit counterparts since they are tax exempt. However, Norton and Staiger [20] find no difference between not-for-profit and for-profit hospitals in provision of uncompensated care once they control for hospital location. However, competition leads to higher rates of decrease in uncompensated care in California [21]. Moreover, not-for-profits act more like for-profits in their presence [22, 23]. Vast majority of not-for-profit hospitals in California provide community services more than the tax subsidies they receive but nearly 20% of not-for-profits constantly do not provide community services more than the tax subsidies they receive [24]. Moreover, they do not provide more socially beneficial activities than for-profits when they have higher market power [25]. Hence, there seems to be a divide among not-for-profit hospitals supporting Hirth’s mixture hypothesis [6, 7].

Hospital markets in the US had become concentrated over time from 1987 to 2002 [26] and the introduction of the Affordable Care Act (ACA) triggered a new wave of hospital consolidation to protect and strengthen market positions and improve operational efficiency [27]. Many commentators believe competition can help curb surging health care expenditures in the US. Recently, the Hospital Competition Act of 2019 was introduced in the US to reduce health care spending by promoting competition among hospitals. However, the evidence on the effects of hospital competition on quality is mixed and highly context dependent.^2^ Moreover, the effect of hospital competition on treatment choices is highly neglected. If higher competition leads to aggressive treatments by hospitals to attract patients, promoting competition may lead to higher health care costs contrary to the aim of the proposed policies. In this work, we will focus on being member of a hospital system and level of competition faced by the hospital to examine hospital ownership types considering treatment choices of insured and uninsured patients.

We assume that a decision is made by the hospital, denoted *h*, between providing the procedure (diagnostic or therapeutic) as the treatment or not providing it, denoted *i* = {0, 1}, where treatment takes value 0 if the procedure is not performed and takes value 1 if the procedure is performed. As an example, routine chest X-ray can be performed during a hospital stay to a patient, denoted *j* = {*insured*, *uninsured*}, admitted with simple pneumonia and pleurisy older than 17 years of age, with complication or comorbidity (diagnosis related group (DRG) 89). Payment to the hospital, *P*_*j*_ does not depend on whether routine chest X-ray is performed or not since hospital payment depends on the DRG of the patient. However, *P*_*j*_ depends on whether the patient is insured or not and performing a chest X-ray costs *C*. We assume that *P*_*insured*_ > *C* and *P*_*uninsured*_ = 0. Furthermore, hospital *h* derives utility *α*_*h*_, which is nonnegative and increasing in the level of altruism of the hospital, from according the procedure as the treatment to the patient. Therefore, the utility hospital *h* obtains from treatment choice *i* to patient *j* is as follows:

$$ {U}_{hij}=\left\{\begin{array}{c}{P}_j-C+{\alpha}_h,i=1\\ {}\ 0,i=0\end{array}\right. $$

Hospitals maximize the sum of utilities they obtain from treating patients subject to their budget constraint, denoted

$$ \sum \limits_{i=1}\left({P}_j-C\right)\ge {F}_h $$

where *F*_*h*_ includes all costs including administrative costs other than marginal cost of according the procedure, and allowed to depend on system membership of the hospital, which is argued to improve operational efficiency [27]. Not-for-profit hospitals are expected to have higher *α* compared to their for-profit counterparts and system hospitals are expected to have lower *F* than system nonmember hospitals. Therefore, we would expect not-for-profits to be more likely to accord the procedure as the treatment to uninsured patients than for-profits. System member hospitals with a positive *α* should be more likely to accord the procedure to uninsured patients than system nonmember hospitals. Under the assumption of for-profit hospitals’ engagement in cream skimming behavior, which is shown in multiple settings [36, 37], not-for-profit hospitals facing high for-profit competition will have a lower share of insured patients and a tighter budget constraint than not-for-profit hospitals facing low for-profit competition. Hence not-for-profit hospitals facing high for-profit competition will be less likely to accord the procedure as the treatment to uninsured patients than not-for-profits facing low for-profit competition even if not-for-profit hospitals facing both high for-profit competition and low for-profit competition have the same level of altruism. To sum up, ownership type of hospitals, system membership and hospital competition may affect the treatment choices of hospitals according to our model.

This work contributes to both hospital ownership literature and hospital competition literature by examining how hospitals differ by ownership type and system membership in terms of the treatment choices of insured and uninsured patients, considering different levels of competition faced from hospitals of different ownership types. We consider all of the procedures performed in the hospitals to obtain a general understanding of the relationship between hospital ownership, system membership, competition and treatment choices. We measure the competition hospitals face separately for each procedure category, utilizing a distance-weighted approach. We employ a logit model and use hospital inpatient records from 11 states from 2004 to 2005 to focus on the pre-ACA, pre-recession years, when close to half of the hospitals in the US were not part of a hospital system.

## Methods

### Data

We employ two datasets in the analysis. Patient records are obtained from State Inpatient Databases (SID) for eleven states (Arizona, Arkansas, Florida, Iowa, Massachusetts, Maryland, New Jersey, New York, Rhode Island, Washington, and Wisconsin), Healthcare Cost and Utilization Project (HCUP), Agency for Healthcare Research and Quality [38], from 2004 to 2005, include the patient characteristics needed to estimate the probability of according the procedures by hospitals but do not include patient identifiers. SID is an all-payer inpatient care database in the US. It contains all discharge data from participating states. General medical and surgical hospitals are used in the analysis. Hospital characteristics of all hospitals are obtained from the second dataset, American Hospital Association (AHA) Annual Survey [39].

53% of hospitals were in a health system during our study period, where 72% of for-profits, 54% of not-for-profits and 37% of government hospitals were members of a system. Especially with the introduction of ACA, hospitals’ participation rate in systems increased considerably to cut on administrative costs. According to AHA hospital statistics 2020 edition, 67% community hospitals in the US were in a system in 2018 [40]. Using data from pre-ACA and pre-recession years allows us to exploit the higher variation in system membership during this period and examine health care markets while they remained considerably stable.

### Outcome variable

In this paper, we examine the relationship between treatment choices and hospital ownership type, system membership and competition. Assuming that a patient can be treated with a procedure, the outcome variable is the binary variable, which takes value of one if patient is accorded the procedure during her hospital stay as the treatment. Instead of focusing on a specific diagnosis and possible treatments for that specific diagnosis, we consider all of the procedures accorded at hospitals, aggregated into clinically meaningful and relatively homogenous mutually exclusive categories,^3^ to have a general understanding of the relationship between treatment choices and hospital ownership type, system membership, and competition. We use computer classification software (CCS) procedural groups, which are developed as part of the HCUP, to examine treatments in the analysis. There are 231 procedural groups (both diagnostic and therapeutic) and up to six procedural CCS groups are reported for each patient record in SID. Since it is not feasible to define target patient groups for all of the procedures reliably using expert opinion or medical literature, we turn to our dataset to determine the target patients for procedures. To obtain relatively homogenous target patient groups, we use DRGs.^4^ We define target patient records for each procedural group as the records with the most frequent three DRGs unless the share of DRG in the group of records with the CCS procedural group as the primary procedure is less than 2%. The interaction of target procedural group and DRGs are included to control for the differences in the necessity of according different procedures to different diagnostic groups. For example, the most frequent three DRGs for routine chest X-ray (CCS 183) are simple pneumonia and pleurisy for age > 17, with complication or comorbidity (DRG 89), heart failure and shock (DRG 127), and chest pain (DRG 143). Since the need to get a chest X-ray is expected to be different for a pneumonia patient and a patient with heart failure or chest pain, we need to control for diagnosis for each treatment. Hence, inclusion of the interactions of target procedural groups and DRGs enables us to include all of the procedures in our analysis. Moreover, one patient record, which is in the target procedural group of more than one procedure, will appear more than once in the dataset but the values CCS procedural groups and DRG interactions take will be different for each case. For instance, a patient older than 17 years of age with simple pneumonia and pleurisy with complication or comorbidity (DRG 89), can get a routine chest X-ray (CCS 183), or chest CT scan (CCS 178), or both or none. This patients’ record will appear separately for routine chest X-ray and chest CT scan. For chest X-ray, from the DRG-procedural group interactions, only the dummy for DRG89xCCS183 will take value 1, whereas for chest CT scan, only DRG89xCCS178 will take value 1. Hence, inclusion of patient records with multiple target procedures does not distort the empirical estimates as a result of inclusion of interactions of target procedural group and DRGs. Furthermore, procedures are weighted by the size of their target patient group as a consequence of selection of target patient records for procedural groups in the analysis, which is preferable to assigning equal weights to procedures when comparing the treatment choices of hospitals in general. Procedure groups and target DRGs used in the analysis are reported in Additional file 1: Table A1.

### Measure of hospital competition

Herfindahl Hirschman Index is frequently used when analyzing competition. However, it has been shown that close-by hospitals compete with each other over quality, but not with hospitals far away [41]. Therefore, it is important to consider the distance between hospitals when examining the effects of hospital competition.

We use a distance-weighted method following Horwitz, and Nichols [16, 17] to calculate the competition each hospital faces for each procedural group, which assigns weights by admissions and inversely by distance so that distant hospitals have less importance relative to close hospitals but may still have an effect. We calculate the competition a hospital faces for each procedural group separately using the number of patient records of the target DRGs. Therefore, we can capture different levels of competition for different procedures. For example, a hospital might be facing high competition for cardiac related procedures but low competition for birth related procedures. The measure is explained in detail in Additional file 2.

We allow hospitals within a state to compete with each other. We classify the hospitals as facing high and low competition from for-profit, government and not-for-profit hospitals separately to allow for the effects of competition to differ by ownership type. Since for-profit hospitals are assumed to be expected profit maximizers and government hospitals are supposed to be market output maximizers, a hospital facing the same level of competition from profit maximizers and market output maximizers may act differently depending on its objective. If the competition a hospital faces from an ownership type for a procedural group is in the top 33% for the ownership type and procedural group, the hospital is classified as in a high competition market for that ownership type and procedural group. If the competition a hospital faces from an ownership type for a procedural group is in the bottom 33%, the hospital is classified as in a low competition market for that ownership type and procedural group.^5^ Additionally, we consider the total competition hospitals face from all ownership types and define facing high and low total competition without distinguishing between ownership types of the competing hospitals to test whether only the level of competition faced by the hospital matters irrespective of the ownership types of the competing hospitals. When total competition is considered and hospital competition by ownership types are included as controls, only hospital competition faced from for-profits is statistically significantly associated with treatment choices, therefore we mainly focus on the competition faced from for-profit hospitals in this paper.

### Control variables

We control for health insurance status (insured^6^ vs uninsured), race (white, black, Hispanic, and other race), gender, and age category (less than 18, between 18 and 34, between 35 and 49, between 50 and 64, between 65 and 79, and more than 79) of the patient records. We control for the comorbidities by Charlson indices (17 indicators) to avoid the problems that unobserved severity, which can be correlated with insurance status of patients and ownership types of hospitals, may cause in the analysis. Even though Charlson and Elixhauser indices have been originally developed to predict mortality, several researchers have used them for risk adjustment of other outcomes [19, 42–45]. The main reason is that patients with certain comorbidities usually incur a more intensive treatment which in our study is reflected by the procedures performed. We also include the interactions of being in a target procedural group and DRGs (656 indicators) to account for different treatment needs by DRG. Hospital characteristics that we control for are ownership type, teaching status defined as being a member of council of teaching hospitals, number of nurses per bed, bed size category (number of beds less than 25, between 25 and 99, between 100 and 199, between 200 and 449, more than 449), metropolitan indicator, which denotes whether the hospital is located in a metropolitan statistical area, and system indicator, which denotes whether hospital is a member of a multihospital or a diversified single hospital system as defined by AHA. Year and state fixed effects are included as well.

### Descriptive statistics

Table 1 reports the summary statistics for all observations, and observations from hospitals facing high and low total competition, and high and low for-profit competition respectively. There are around 78 million observations in the dataset when the target patient records for each procedural group are included.^7^ Approximately one third of the observations fall into high competition markets by definition. 62% of the records are from system hospitals. 68 and 75% of records come from system hospitals in high total competition and high for-profit competition markets, respectively, and the share of records from system hospitals are around 20 percentage points lower in low competition markets than high competition markets. 79.3% of the observations come from not-for-profit hospitals, 11.4% from for-profit hospitals and 9.3% from government hospitals in our dataset.^8^ 1007 out of the 1042 hospitals in our dataset are observed in 2004 and 2005. There are 20 changes in system membership and 9 changes in ownership type. Competition categories changed in 12.3% of hospital-procedure groups in our dataset.

**Table 1:** Summary Statistics for all records and by market competitiveness. Standard deviations are reported in parenthesis

	All	High competition	Low competition	High for-profit competition	Low for-profit competition
System Hospital	0.62	0.68	0.49	0.75	0.55
	(0.49)	(0.47)	(0.50)	(0.43)	(0.50)
Not-for-profit	0.79	0.82	0.78	0.66	0.90
	(0.41)	(0.39)	(0.41)	(0.47)	(0.30)
For-profit	0.11	0.08	0.13	0.22	0.02
	(0.32)	(0.27)	(0.34)	(0.42)	(0.15)
Government	0.09	0.11	0.09	0.12	0.08
	(0.29)	(0.31)	(0.28)	(0.33)	(0.27)
Number of beds	412	574	238	468	369
	(353)	(432)	(167)	(411)	(297)
Number of nurses per bed	1.44	1.53	1.35	1.39	1.44
	(0.51)	(0.53)	(0.51)	(0.49)	(0.49)
Teaching	0.25	0.52	0.04	0.23	0.25
	(0.43)	(0.50)	(0.19)	(0.42)	(0.43)
Metropolitan	0.92	1.00	0.78	0.92	0.92
	(0.26)	(0.00)	(0.42)	(0.26)	(0.27)
Uninsured	0.05	0.06	0.05	0.06	0.05
	(0.22)	(0.23)	(0.22)	(0.23)	(0.22)
Female	0.61	0.61	0.61	0.61	0.62
	(0.49)	(0.49)	(0.49)	(0.49)	(0.49)
White	0.62	0.47	0.74	0.58	0.67
	(0.49)	(0.50)	(0.44)	(0.49)	(0.47)
Black	0.14	0.22	0.08	0.16	0.11
	(0.34)	(0.41)	(0.26)	(0.36)	(0.32)
Hispanic	0.10	0.17	0.05	0.16	0.07
	(0.31)	(0.38)	(0.22)	(0.37)	(0.25)
Number of observations	77,967,689	26,795,506	25,560,311	26,509,222	25,940,642

Number of nurses per bed is lower in high for-profit competition markets than low for-profit competition markets suggesting more profit seeking behavior in high for-profit competition markets. By contrast, number of nurses per bed is higher in high total competition markets than low total competition markets. Around one quarter of records are from teaching hospitals in high and low for-profit competition markets whereas more than half of the records in high total competition markets are from teaching hospitals. On average 5% of the observations are uninsured. The share of white patient records is 11 percentage points higher in high for-profit competition markets than high total competition markets and the share of black patient records is 6 percentage points lower in high for-profit competition markets than high total competition markets. Therefore, summary statistics suggest that for-profit competition is more likely to be associated with profit seeking behavior than total competition.

### Empirical model

We estimate the probability of according the procedure by hospital ownership type, system membership and market competitiveness employing a logit model. Given the hospitals’ utility maximization problem we consider in the background section, treatment choices of hospitals even with the same level of altruism may differ as a result of differences in system membership and the level of competition they face. Furthermore, we would like to determine whether some of the not-for-profits are for-profits in disguise and the conditions under which not-for-profit hospitals act like their for-profit counterparts. We allow for the effect of patient insurance status to differ by hospital ownership type and system membership by including patient insurance status, hospital ownership type and system membership interactions. We also allow the effect of facing high competition to differ by hospital ownership type, system membership, and patient insurance status. Patient records from high and low competition markets for the relevant competition type are included in the analysis. We control for the patient characteristics and hospital characteristics reported as control variables. Heteroscedasticity robust standard errors are clustered at the hospital level so that they are robust to arbitrary serial correlation, which is likely to be present because the probability of a hospital according a procedure is not independent over time.

The equation of estimation examining system membership:

1 $$ E{(PTP)}_{ijt}=F\left({\beta}_0+{\beta}_1{S}_{it}+{\beta}_2{O}_{it}+{\beta}_3{U}_{jt}+{\beta}_4{S}_{it}\ast {U}_{jt}+{\beta}_5{S}_{it}\ast {O}_{it}+{\beta}_6{O}_{it}\ast {U}_{jt}+{\beta}_7{S}_{it}\ast {O}_{it}\ast {U}_{jt}+{\beta}_8{H}_{it}+{\beta}_9{P}_{jt}+{\beta}_{10}p\ast {DRG}_{jt}+{\beta}_{11}{Y}_t\right) $$

The equation of estimation examining system membership and competition:

2 $$ E{(PTP)}_{ijt}=F\left({\beta}_0+{\beta}_1{S}_{it}+{\beta}_2{O}_{it}+{\beta}_3{U}_{j\mathrm{t}}+{\beta}_4{S}_{it}\ast {U}_{jt}+{\beta}_5{S}_{it}\ast {O}_{it}+{\beta}_6{O}_{it}\ast {U}_{jt}+{\beta}_7{S}_{it}\ast {O}_{it}\ast {U}_{jt}+{\beta}_8{HC}_{it}+{\beta}_9{HC}_{it}\ast {S}_{it}+{\beta}_{10}{HC}_{it}\ast {O}_{it}+{\beta}_{11}{HC}_{it}\ast {U}_{it}+{\beta}_{12}{HC}_{it}\ast {S}_{it}\ast {O}_{it}+{\beta}_{13}{HC}_{it}\ast {S}_{it}\ast {U}_{jt}+{\beta}_{14}{HC}_{it}\ast {O}_{it}\ast {U}_{jt}+{\beta}_{15}{HC}_{it}\ast {S}_{it}\ast {O}_{it}\ast {U}_{jt}+{\beta}_{16}{H}_{it}+{\beta}_{17}{P}_{jt}+{\beta}_{18}p\ast {DRG}_{jt}+{\beta}_{19}{Y}_t\right) $$

where *PTP* is the dummy for patient treated with the procedure, *i* represents hospitals, *j* represents patient records and *t* is the year. *S* is the indicator variable for system membership, *O* is a set of indicator variables for not-for-profit and government ownership, *U*_*j*_ is the indicator variable indicating whether patient record *j* belongs to an uninsured patient. *HC* is the indicator variable for facing high competition. We examine total competition and for-profit competition separately. *H* is a vector of hospital characteristics including indicator variables for states. *P* is a vector of patient characteristic variables, *p* is the vector of procedure group dummies and *DRG* is the vector of DRG dummies, and *Y* is an indicator variable for year.

## Results

**Fig. 1:**
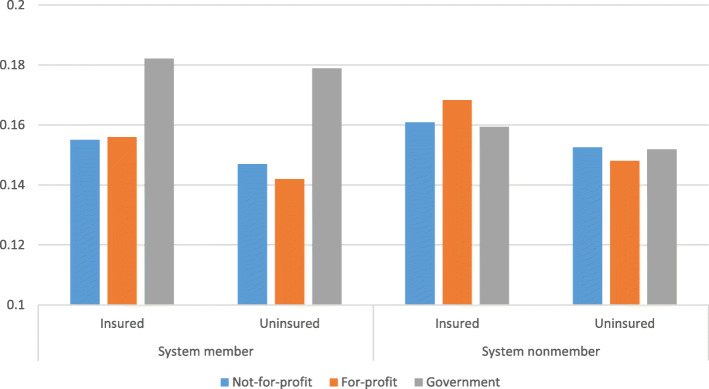
Predicted probabilities of according the procedure by hospital ownership type, system membership, and patient insurance status. Predicted probabilities are calculated as if all of the observations belonged to the same category (as if all of the patients were insured, admitted to system member not-for-profit hospitals, etc.) using estimation results of Table 2, specification IV

Table 2 reports the logistic regression results of the equation of estimation examining system membership (eq. 1). Figure 1 shows the predicted probabilities of according the procedure by hospital ownership type, system membership and patient insurance status. The predicted probabilities are calculated as if all of the patients in our dataset belonged to the same category (for example for the first column of Fig. 1 as if all of the patients were insured and treated at system member not-for-profit hospitals) to visualize the impact of hospital ownership type, system membership, patient insurance status on treatment choices and to eliminate the differences in treatment choices due to differences in the characteristics of the patients admitted at different types of hospitals. On average, an insured patient at a system member government hospital has 4 percentage points higher probability of being accorded the procedure as the treatment than an uninsured patient at a system member for-profit hospital. To examine the statistical significance of the differences among predicted probabilities shown in Fig. 1, we report the odds ratios by patient insurance status, hospital ownership type, and system membership in Table 3. Uninsured patients are statistically significantly less likely to be accorded the procedure than insured patients at not-for-profit and system member for-profit hospitals.^9^ Moreover, there is no statistically significant difference in probability of according the procedure between both system member and nonmember not-for-profit and for-profit hospitals for both uninsured and insured patients supporting Pauly and Redisch’s for-profit in disguise theory [5].

**Table 2:** Logistic Estimation Results

	I	II	III	IV
Not-for-profit	0.012 (0.041)	0.008 (0.041)	−0.090 (0.085)	−0.094 (0.089)
Government	0.163** (0.081)	0.152* (0.081)	−0.111 (0.108)	−0.113 (0.113)
Uninsured		−0.204*** (0.029)		−0.265 (0.224)
Not-for-profit*Uninsured		0.092*** (0.034)		0.156 (0.226)
Government*Uninsured		0.219*** (0.056)		0.165 (0.231)
System			−0.157* (0.087)	−0.158* (0.091)
System*Uninsured				0.072 (0.225)
System*Not-for-profit			0.081 (0.092)	0.082 (0.096)
System*Government			0.438*** (0.167)	0.437** (0.172)
System*Not-for-profit*Uninsured				−0.073 (0.228)
System*Government*Uninsured				−0.010 (0.236)
Number of observations	77,606,823	77,606,823	77,606,823	77,606,823

*Notes.* Year and state fixed effects, age category indicators, weighted charlson indices, female and race indicators, bed size categories, number of nurses per bed, teaching and metropolitan indicators and target ccs DRG interaction indicators are included as controls in the regressions. Heteroscedasticity robust standard errors clustered at the hospital level are reported in parentheses. ***p < 0.01, **p < 0.05, *p < 0.1

**Table 3:** Odds ratios of according the procedure by (a) patient insurance status, (b) hospital ownership type, and (c) system membership

**(a) Uninsured/Insured**		Not-for-profit	For-profit	Government
System member		0.90*** (0.85, 0.94)	0.82*** (0.79, 0.87)	0.96 (0.88, 1.06)
System nonmember		0.90*** (0.84, 0.96)	0.77 (0.50, 1.19)	0.91* (0.82, 1.01)
**(b)**		Not-for-profit/ For-profit	Government/ For-profit	Not-for-profit/ Government
System member	Insured	0.99 (0.91, 1.08)	1.38** (1.07, 1.79)	0.71*** (0.56, 0.91)
	Uninsured	1.07 (0.96, 1.19)	1.62*** (1.26, 2.07)	0.66*** (0.52, 0.84)
System nonmember	Insured	0.91 (0.76, 1.08)	0.89 (0.72, 1.11)	1.02 (0.88, 1.18)
	Uninsured	1.06 (0.72, 1.57)	1.05 (0.71, 1.57)	1.01 (0.87, 1.17)
**(c) System member/Nonmember**		Not-for-profit	For-profit	Government
Insured		0.93** (0.87, 0.99)	0.85* (0.71, 1.02)	1.32** (1.01, 1.74)
Uninsured		0.93 (0.83, 1.03)	0.92 (0.62, 1.35)	1.41*** (1.09, 1.82)

*Notes.* Tests are performed using estimation results of Table 2, specification IV. 95% confidence intervals of the odds ratios are reported in parentheses. ***p < 0.01, **p < 0.05, *p < 0.1

**Fig. 2:**
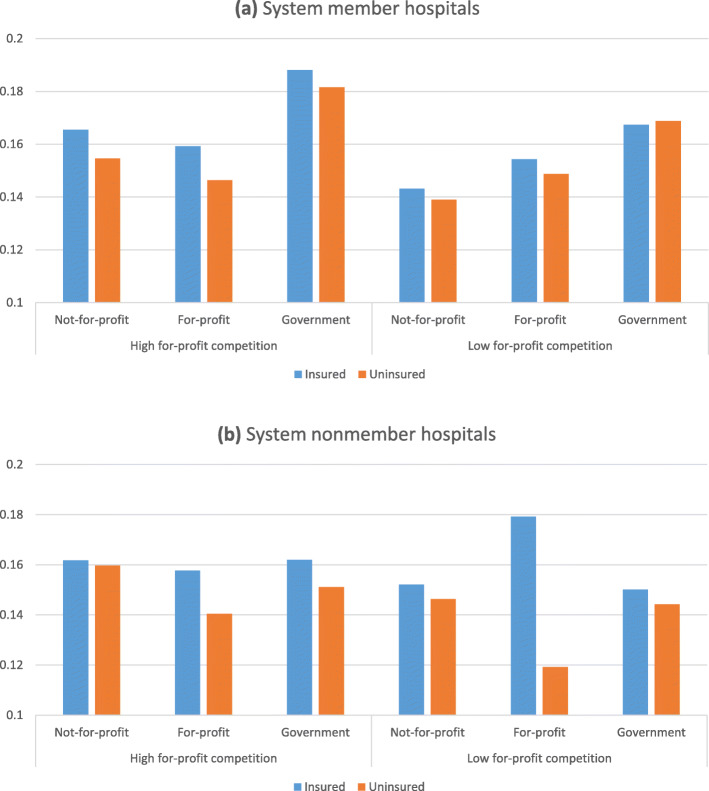
Predicted probabilities of according the procedure by hospital ownership type, market competitiveness, patient insurance status at (**a**) system member and (**b**) system nonmember hospitals. Predicted probabilities are calculated as if all of the observations belonged to the same category (as if all of the patients were insured, admitted to system member not-for-profit hospitals facing high for-profit competition, etc.) using estimation results of Table 3, specification VII

Table 4 reports the logistic results with the level of competition faced by hospitals (eq. 2).^10^ Figure 2 shows that on average uninsured patients are 6 percentage points less likely to be accorded the procedure as the treatment than insured patients at system nonmember for-profit hospitals facing low for-profit competition. Uninsured patients are on average 7% less likely to be accorded the procedure as the treatment at system member not-for-profit hospitals facing high for-profit competition than insured patients. Table 5 reports the odds ratios by patient insurance status, hospital ownership type, system membership and the level of for-profit competition faced by the hospitals. Treatment choice probabilities do not statistically significantly differ for both insured and uninsured patients at 5% level among system member not-for-profit and for-profit hospitals and nonmember hospitals facing high for-profit competition as reported in Table 5(b). However, when we consider nonmember hospitals facing low for-profit competition, for-profits are more likely to accord the procedure to insured than not-for-profits (odds ratio: 0.71) whereas not-for-profits are more likely to accord the procedure to uninsured than for-profits (odds ratio: 1.51), both differences statistically significant at 1% level.

**Table 4:** Logistic Estimation Results with Competition

	V^a^	VI^b^	VII^b^	VIII^c^
Not-for-profit	−0.249 (0.171)	−0.362*** (0.101)	−0.339*** (0.093)	−0.248** (0.104)
Government	−0.226 (0.183)	−0.392*** (0.143)	−0.366*** (0.137)	−0.314** (0.145)
Uninsured	−0.310 (0.366)	−0.823*** (0.087)	−0.828*** (0.082)	−0.702*** (0.162)
Not-for-profit*Uninsured	0.120 (0.368)	0.742*** (0.098)	0.750*** (0.094)	0.616*** (0.166)
Government*Uninsured	0.274 (0.371)	0.740*** (0.126)	0.746*** (0.122)	0.664*** (0.174)
System	−0.248 (0.179)	−0.342*** (0.120)	−0.309*** (0.113)	−0.156 (0.127)
System*Uninsured	0.133 (0.368)	0.748*** (0.118)	0.752*** (0.115)	0.611*** (0.174)
System*Not-for-profit	0.245 (0.183)	0.213 (0.131)	0.187 (0.126)	0.098 (0.138)
System*Government	0.078 (0.231)	0.559*** (0.175)	0.533*** (0.170)	−0.116 (0.183)
System*Not-for-profit*Uninsured	−0.096 (0.371)	−0.726*** (0.135)	−0.734*** (0.133)	−0.612*** (0.183)
System*Government*Uninsured	0.189 (0.419)	−0.648*** (0.154)	−0.653*** (0.151)	−0.406* (0.220)
High for-profit competition	0.259*** (0.051)	−0.315* (0.165)	−0.265* (0.157)	−0.294* (0.169)
Medium for-profit competition	0.184*** (0.032)			
High not-for-profit competition	0.043 (0.064)			
Medium not-for-profit competition	0.017 (0.046)			
High government competition	0.021 (0.057)			
Medium government competition	0.056 (0.039)			
High total competition	−0.257 (0.185)	0.059 (0.043)		
Medium total competition		0.061* (0.037)		
High competition* Not-for-profit	0.372** (0.190)	0.432*** (0.165)	0.392** (0.159)	0.502*** (0.174)
High competition* Government	0.188 (0.245)	0.463** (0.215)	0.421** (0.210)	0.616*** (0.233)
High competition* Uninsured	0.134 (0.401)	0.584*** (0.172)	0.593*** (0.170)	0.615*** (0.196)
High competition* Not-for-profit*Uninsured	−0.068 (0.407)	−0.532*** (0.194)	−0.542*** (0.193)	−0.595*** (0.222)
High competition* Government*Uninsured	−0.222 (0.420)	−0.647*** (0.200)	−0.654*** (0.197)	−0.752*** (0.222)
System*High competition	0.203 (0.197)	0.368** (0.171)	0.330** (0.167)	0.266 (0.178)
System*High competition* Not-for-profit	−0.362* (0.207)	−0.203 (0.187)	−0.162 (0.182)	−0.350* (0.199)
System*High competition* Government	0.466 (0.303)	−0.285 (0.284)	−0.241 (0.280)	0.187 (0.288)
System*High competition* Uninsured	−0.207 (0.410)	−0.680*** (0.190)	−0.689*** (0.189)	−0.713*** (0.208)
System*High competition* Not-for-profit*Uninsured	0.218 (0.418)	0.550** (0.219)	0.560** (0.220)	0.655*** (0.242)
System*High competition* Government*Uninsured	−0.073 (0.473)	0.652*** (0.228)	0.658*** (0.227)	0.626** (0.266)
Number of observations	51,752,777	52,103,007	52,103,007	51,837,255

^a^Total competition measured at hospital-ccs level

^b^For-profit competition measured at hospital-ccs level

^c^For-profit competition measured at the hospital level

*Notes.* Year and state fixed effects, age category indicators, weighted Charlson indices, female and race indicators, bed size categories, number of nurses per bed, teaching and metropolitan indicators and target ccs DRG interaction indicators are included as controls in the regressions. Heteroscedasticity robust standard errors clustered at the hospital level are reported in parentheses. ***p < 0.01, **p < 0.05, *p < 0.1

**Table 5:** Odds ratios of according the procedure by (a) patient insurance status, (b) hospital ownership type, (c) system membership, and (d) the level of for-profit competition faced by the hospital

**(a) Uninsured/Insured**			Not-for-profit	For-profit	Government
HFC	System member		0.87*** (0.81, 0.93)	0.84*** (0.80, 0.89)	0.93 (0.81, 1.06)
	System nonmember		0.97 (0.84, 1.13)	0.79 (0.59, 1.06)	0.87*** (0.79, 0.95)
LFC	System member		0.94 (0.86, 1.03)	0.93 (0.79, 1.08)	1.02 (0.94, 1.10)
	System nonmember		0.92* (0.84, 1.01)	0.44*** (0.37, 0.51)	0.92 (0.77, 1.10)
**(b)**			Not-for-profit/ For-profit	Government/ For-profit	Not-for-profit/ Government
HFC	System member	Insured	1.08 (0.97, 1.20)	1.42* (0.98, 2.04)	0.76 (0.53, 1.10)
		Uninsured	1.12* (0.98, 1.28)	1.56*** (1.13, 2.15)	0.72** (0.52, 1.00)
	System nonmember	Insured	1.05 (0.82, 1.36)	1.06 (0.76, 1.47)	1.00 (0.77, 1.29)
		Uninsured	1.30 (0.92, 1.83)	1.16 (0.83, 1.63)	1.12 (0.85, 1.48)
LFC	System member	Insured	0.86* (0.74, 1.00)	1.18 (0.95, 1.47)	0.73*** (0.60, 0.87)
		Uninsured	0.87 (0.68, 1.11)	1.30* (0.97, 1.73)	0.67*** (0.53, 0.85)
	System nonmember	Insured	0.71*** (0.59, 0.85)	0.69*** (0.53, 0.91)	1.03 (0.82, 1.28)
		Uninsured	1.51*** (1.25, 1.82)	1.46*** (1.10, 1.95)	1.03 (0.78, 1.36)
**(c) System member/Nonmember**			Not-for-profit	For-profit	Government
HFC	Insured		1.05 (0.91, 1.20)	1.02 (0.80, 1.30)	1.37 (0.89, 2.10)
	Uninsured		0.94 (0.74, 1.18)	1.09 (0.82, 1.45)	1.46** (1.01, 2.11)
LFC	Insured		0.88** (0.80, 0.98)	0.73*** (0.59, 0.92)	1.25* (0.97, 1.61)
	Uninsured		0.90 (0.76, 1.07)	1.56*** (1.20, 2.02)	1.38** (1.01, 1.88)
**(d) HFC/LFC**	System member			System nonmember	
	Insured		Uninsured	Insured	Uninsured
Not-for-profit	1.34*** (1.19, 1.52)		1.24** (1.05, 1.47)	1.14 (0.96, 1.35)	1.19 (0.93, 1.53)
For-profit	1.07 (0.91, 1.25)		0.97 (0.77, 1.22)	0.77* (0.56, 1.04)	1.39* (0.99, 1.95)
Government	1.28 (0.91, 1.80)		1.16 (0.86, 1.57)	1.17 (0.86, 1.58)	1.10 (0.80, 1.51)

*Notes.* Tests are performed using estimation results of Table 4, specification VII. HFC and LFC denote facing high for-profit competition and low for-profit competition respectively. 95% confidence intervals of the odds ratios are reported in parentheses. ***p < 0.01, **p < 0.05, *p < 0.1

When hospitals face high for-profit competition, treatment choices do not statistically significantly differ by system membership for not-for-profit and for-profit hospitals. Nonmember for-profits facing low for-profit competition are very aggressive in treatment choices of insured compared to uninsured patients (odds ratio and 95% confidence interval for uninsured vs insured is 0.44 (0.37, 0.51)). Furthermore, system member not-for-profit hospitals, which account for 52.3% of observations in our dataset are statistically significantly more likely to accord the procedure to both insured and uninsured patients in high for-profit competition markets than low for-profit competition markets with odds ratios (95% confidence intervals) of 1.34 (1.19, 1.52) and 1.24 (1.05, 1.47) for insured and uninsured patients respectively.

In specifications V, VI, and VII, competition faced by a hospital is calculated separately by CCS procedural group, whereas in specification VIII, we used the aggregate competition measure, which does not distinguish by target procedural groups while calculating the competition a hospital faces. Estimated effects of hospital competition considerably differs both qualitatively and quantitatively when the competition a hospital faces is calculated at the hospital level without distinguishing between target procedural groups. With the aggregate competition measure, system membership is found not to have any statistically significant effect on treatment choices for insured patients at hospitals facing both high and low for-profit competition. However, system member not-for-profits and for-profits are statistically significantly less likely to accord the procedure as the treatment to insured patients than nonmember hospitals when competition is measured at the CCS procedural group level. Therefore, we would have missed the effect of system membership and competition if we had only measured hospital competition at the hospital level without considering different target patient groups for procedures.^11^

## Discussion

This study examines treatment choices considering hospital ownership type, system membership, insurance status of patients and the level of competition faced by hospitals. We include all of the procedures in the analysis, controlling for interactions of target procedural group and DRGs, to obtain a general understanding of the relationship between treatment choices, hospital ownership type, system membership and market competitiveness. We use a distance-weighted measure calculated separately for each procedural group for precise measurement of hospital competition. Our aim is to assess whether patients are treated differently, without conditioning on treatment availability at the hospitals. We do not assess the appropriateness of the treatments or make claims regarding over/under treatment of patients. Given that we examine all of the procedures accorded at hospitals, it is not possible to determine the appropriate level of according the procedure. Therefore, both insured and uninsured patients might be overtreated or undertreated. Moreover, more aggressive treatments do not necessarily lead to better outcomes for the patients. Long-term outcomes for the Medicare population were not better in higher intensity U.S. regions [47–49], or in high-intensity compared with low-intensity academic medical centers [50]. Complex surgery does not improve outcomes in patients with advanced-stage ovarian cancer when accounting for other confounding influences [51]. Additionally, more aggressive procedure leads to a higher mortality rate in patients with acute myocardial infarction and cardiogenic shock [52–54]. However, our analysis shows that uninsured patients are more likely to be undertreated than insured patients especially under some conditions. We show that ownership type of competing hospitals are important as well as the level of competition and the competition faced from for-profit hospitals has a statistically significant effect on treatment choices. Treatment choices of system member and nonmember hospitals facing low for-profit competition differ significantly, whereas treatment choices at not-for-profit and for-profit hospitals facing high for-profit competition do not differ by system membership.

Previous empirical work examining market competitiveness found that not-for-profits in relatively high for-profit markets were engaged in more profit seeking behavior such as provision of profitable services [17], avoiding unprofitable patients [55, 56], spending less on admitted cardiac patients [30], and being more responsive to profit-making opportunities [22, 23, 57]. We find that treatment choices of system member not-for-profit hospitals facing both high and low for-profit competition and nonmember not-for-profit hospitals facing high for-profit hospital competition are not statistically significantly different from their for-profit counterparts, supporting that *not-for-profits participating in hospital systems and nonmember not-for-profits facing high for-profit competition are for-profits in disguise* as proposed by Pauly and Redisch [5]. System nonmember not-for-profit hospitals facing low for-profit competition are more likely to accord the procedure to uninsured and less likely to accord to insured patients than their for-profit counterparts are. Moreover, they are not statistically significantly different from government hospitals in terms of treatment choices of both insured and uninsured patients, supporting that *not-for-profits not participating in hospital systems facing low for-profit competition are market output maximizers* as proposed by Weisbrod [4]. Hence, system membership is an important determinant of profit seeking behavior of not-for-profits in addition to competitiveness of the market.

Even though hospital competition does not have an effect on socioeconomic health care inequality in England [58], this does seem to be the case in the US. System member not-for-profit and for-profit hospitals, and system nonmember government hospitals facing high for-profit competition are significantly more likely to accord the procedure to insured patients than uninsured, whereas among the hospitals facing low for-profit competition, only system nonmember for-profits are significantly more likely to accord the procedure to insured than uninsured. Hence, higher for-profit competition is associated with higher inequality in treatment choices of insured and uninsured in the US. Moreover, not-for-profit and for-profit hospitals are less likely to accord the procedure to uninsured patients than to insured patients. Therefore, expanding health care coverage for the uninsured will lead to lower inequality in the treatments accorded to patients admitted to the hospitals in addition to improving access to health care.

Our study has several limitations. 1. We use state inpatient databases for 11 states from 2004 to 2005 rather than all of the U.S. states for a longer time period covering post-ACA period, which was infeasible because of our budget constraint. The states used in the analysis are geographically diverse, represent 28% of the U.S. population, and are selected from a subset of states that provide hospital identifiers, which enables us to obtain the hospital characteristics from AHA. 2. Due to the limited time period, there are few changes in hospital ownership type and system membership from 2004 to 2005. Therefore, the effects of hospital ownership type and system membership on treatment choices are estimated based mainly on cross-sectional variation. The variation in the level of competition faced by the hospitals from 2004 to 2005 is considerably higher than the variation in hospital ownership type and system membership over time but the estimated effects of competition are mostly based on cross-sectional variation in competition. 3. Our results are based on pre-ACA period. To examine the effect of system membership and competition, which changed as a result of ACA, it would be ideal to do a pre-ACA vs post-ACA analysis. 4. We observe the DRG in effect on discharge date, which might be different from the DRG when hospitals make treatment decisions. Or hospitals may be making treatment decisions based on the expected DRG on discharge. We assume that the ability to predict the DRG at discharge is not correlated with ownership type, system membership or the level of competition hospitals face. 5. We do not observe relative profitability of DRGs, hence we cannot perform subgroup analysis for profitable and unprofitable DRGs, which would lead to a clearer understanding of profit seeking behavior of hospitals. Hence, our conclusions regarding profit seeking behavior of hospitals hinges on the unprofitability of uninsured patients. 6. We do not observe whether a certain treatment was recommended by the hospital and rejected by the patient due to financial or other concerns. If uninsured patients are more likely to be concerned about financial consequences of their treatments at hospitals of certain ownership types, the differences in accordance probabilities of procedures may be partly attributed to the decision of the patient rather than hospital. If that is the case, one may argue that hospitals of certain ownership types are more likely to convince the patients to receive the procedure as the treatment due to differences in hospitals’ attitudes towards the patients of different insurance status across ownership types, which indicates difference in profit seeking behavior among ownership types.

## Conclusions

We show that system membership and the competition hospitals face from for-profits are significantly associated with treatment choices of hospitals as well as hospital ownership type and patients’ insurance status. Our results support that *not-for-profit hospitals facing low for-profit competition, which are not member of hospital systems, are market output maximizers* just like government hospitals, whereas *the not-for-profits facing high for-profit competition and system member not-for-profits are for-profits in disguise*. Hence, system membership is an important characteristic to consider in addition to market competitiveness when tax exemption of not-for-profits are revisited. Moreover, promoting hospital competition may lead to higher health care costs due to more aggressive treatment choices by hospitals in contrary to the aim of the Hospital Competition Act of 2019. Therefore, potential cost increasing implications of higher competition should be taken into account by policymakers while regulating hospital markets.

## Supplementary Information

**Additional file 1: Table A1.** Selected DRGs for procedure groups defined by ccs procedure.

**Additional file 2:** Distance Weighted Measure of Hospital Competition.

## Data Availability

State inpatient databases used in the analysis are available for purchase from Agency for Healthcare Research and Quality, Healthcare Cost and Utilization Project (HCUP) (https://www.hcup-us.ahrq.gov/tech_assist/centdist.jsp) and American Hospital Association Annual Survey is available for purchase through https://www.ahadata.com, so they are not publicly available. The code used in the analysis is available upon request from the authors.

## Abbreviations

ACA: Affordable Care Act
AHA: American Hospital Association
CCS: computer classification software
DRG: diagnosis-related group
HCUP: Healthcare Cost and Utilization Project
SID: State Inpatient Databases
US: United States

## Footnotes

1 Spika and Zweifel propose a payment system, which promotes bargaining to hinder upcoding [13].

2 Mortality rates for Medicare acute myocardial infarction patients are higher in highly concentrated markets [28] and hospital entry leads to improved quality for heart bypass surgery [29]. In the areas with for-profit hospitals, expenditures are lower and health outcomes are not different for Medicare patients [30]. Increased competition for health maintenance organization patients leads to higher quality of care whereas increased competition for Medicare patients leads to lower quality of care when pneumonia and acute myocardial infarction patients in California are examined [31]. On the other hand, market concentration does not have an effect on mortality from all causes for Medicare patients [32]. A 2006 reform introduced to promote competition among hospitals in the English National Health Services led to higher improvement in outcomes in less concentrated markets than more concentrated markets for acute myocardial infarction patients [33, 34]. However, hospital market concentration does not have any significant effect on the outcome of elective primary hip replacement in England [35].

3 For example, 21 ICD-9-CM procedure codes (35.00–35.04, 35.10–35.28, 35.96, 35.99) are grouped as “heart valve procedures” (CCS 43) and 14 ICD-9-CM procedure codes (36.10–36.32, 36.39) are grouped as “coronary artery bypass graft” (CCS 44).

4 Payment to the hospital is independent of whether the procedure is accorded as the treatment or not in this setting. Results are robust when CCS diagnostic groups are used instead of DRGs.

5 Results are robust to alternative cutoffs for high and low competition markets. We used 33% for the sake of symmetry.

6 We have grouped Medicare, Medicaid and privately insured patients as insured for tractability of the results. Results are robust to examining patient records by insurance types.

7 There are 17,784,031 patient records in SID datasets, of which 79.5% are from not-for-profit hospitals, 11% are from for-profit hospitals and 9.5% are from government hospitals. 11% of the patient records of government hospitals belongs to uninsured patients, whereas 5 and 6% of patients are uninsured at not-for-profit and for-profit hospitals, respectively.

8 The distribution of observations across hospitals used in the analysis are very similar to the distribution of patient records.

9 Odds ratios for uninsured vs insured patients are 0.90 and 0.82 for not-for-profit and system member for-profit hospitals respectively.

10 In specification V, total competition without distinguishing by hospital ownership type is considered controlling for high and medium levels of for-profit, not-for-profit and government competition. Because only high and medium for-profit competition indicators are statistically significantly associated with the probability of according the procedure, we used high for-profit competition indicator in specification VI controlling for the level of total competition, which turned out to be insignificant at 5% level. Hence, we excluded high and medium total competition indicators in specification VII, which is used to calculate the predicted probabilities shown in Figure 2 and odds ratios reported in Table 5.

11 Similarly, Herfindahl Hirschman Index calculated at the hospital level is shown to be a very poor proxy for true market concentration in Germany [46].
