# Supplementary material for: What drives different treatment choices? Investigation of hospital ownership, system membership and competition
Source: Health Econ Rev. 2021 Feb 16;11:6. doi: 10.1186/s13561-021-00305-3 (PMC7885748; doi:10.1186/s13561-021-00305-3)
Supplement: Supplementary file 1 — Additional file 1: Table A1. Selected DRGs for procedure groups defined by ccs procedure. [file 13561_2021_305_MOESM1_ESM.docx]

# **Appendix A**

**Table A1.** Selected DRGs for procedure groups defined by ccs procedure.

| ccs# | ccs definition | DRGs |
| --- | --- | --- |
| 1 | Incision and excision of CNS | 1, 2, 3 |
| 2 | Insertion; replacement; or removal of extracranial ventricular shunt | 1, 3 |
| 3 | Laminectomy; excision intervertebral disk | 500, 499, 498 |
| 4 | Diagnostic spinal tap | 21, 422, 389 |
| 5 | Insertion of catheter or spinal stimulator and injection into spinal canal | 243, 373, 492 |
| 6 | Decompression peripheral nerve | 6, 8, 228 |
| 7 | Other diagnostic nervous system procedures | 1, 10, 11 |
| 8 | Other non-OR or closed therapeutic nervous system procedures | 25, 24, 243 |
| 9 | Other OR therapeutic nervous system procedures | 1, 4, 234 |
| 10 | Thyroidectomy; partial or complete | 290 |
| 11 | Diagnostic endocrine procedures | 300, 289, 301 |
| 12 | Other therapeutic endocrine procedures | 289, 286, 394 |
| 13 | Corneal transplant | 42, 40, 477 |
| 14 | Glaucoma procedures | 42, 477, 38 |
| 15 | Lens and cataract procedures | 39, 477, 36 |
| 16 | Repair of retinal tear; detachment | 36, 477, 48 |
| 17 | Destruction of lesion of retina and choroid | 48, 386, 36 |
| 18 | Diagnostic procedures on eye | 42, 37, 40 |
| 19 | Other therapeutic procedures on eyelids; conjunctiva; cornea | 40, 42, 477 |
| 20 | Other intraocular therapeutic procedures | 42, 36, 40 |
| 21 | Other extraocular muscle and orbit therapeutic procedures | 37, 477, 40 |
| 22 | Tympanoplasty | 55, 54, 62 |
| 23 | Myringotomy | 62, 477, 61 |
| 24 | Mastoidectomy | 53, 54, 468 |
| 25 | Diagnostic procedures on ear | 73, 55, 74 |
| 26 | Other therapeutic ear procedures | 55, 49, 73 |
| 27 | Control of epistaxis | 66, 134, 63 |
| 28 | Plastic procedures on nose | 56, 55, 72 |
| 29 | Dental procedures | 187, 168, 169 |
| 30 | Tonsillectomy and/or adenoidectomy | 60, 443, 59 |
| 31 | Diagnostic procedures on nose; mouth and pharynx | 64, 73, 57 |
| 32 | Other non-OR therapeutic procedures on nose; mouth and pharynx | 185, 390, 73 |
| 33 | Other OR therapeutic procedures on nose; mouth and pharynx | 50, 57, 52 |
| 34 | Tracheostomy; temporary and permanent | 483, 482 |
| 35 | Tracheoscopy and laryngoscopy with biopsy | 73, 391, 390 |
| 36 | Lobectomy or pneumonectomy | 75 |
| 37 | Diagnostic bronchoscopy and biopsy of bronchus | 76, 82, 89 |
| 38 | Other diagnostic procedures on lung and bronchus | 75, 468, 400 |
| 39 | Incision of pleura; thoracentesis; chest drainage | 82, 85, 127 |
| 40 | Other diagnostic procedures of respiratory tract and mediastinum | 76, 82, 77 |
| 41 | Other non-OR therapeutic procedures on respiratory system | 82, 144, 101 |
| 42 | Other OR therapeutic procedures on respiratory system and mediastinum | 75, 76, 55 |
| 43 | Heart valve procedures | 105, 104, 112 |
| 44 | Coronary artery bypass graft (CABG) | 107, 109, 108 |
| 45 | Percutaneous transluminal coronary angioplasty (PTCA) | 116, 112 |
| 46 | Coronary thrombolysis | 122, 121, 132 |
| 47 | Diagnostic cardiac catheterization; coronary arteriography | 125, 124, 121 |
| 48 | Insertion; revision; replacement; removal of cardiac pacemaker or cardioverter/defibrill | 116, 104, 105 |
| 49 | Other OR heart procedures | 112, 110, 108 |
| 50 | Extracorporeal circulation auxiliary to open heart procedures | 105, 104, 385 |
| 51 | Endarterectomy; vessel of head and neck | 5 |
| 52 | Aortic resection; replacement or anastomosis | 110, 111, 468 |
| 53 | Varicose vein stripping; lower limb | 119, 477 |
| 54 | Other vascular catheterization; not heart | 416, 277, 89 |
| 55 | Peripheral vascular bypass | 478, 479, 110 |
| 56 | Other vascular bypass and shunt; not heart | 191, 110, 5 |
| 57 | Creation; revision and removal of arteriovenous fistula or vessel-to-vessel cannula | 120, 315, 468 |
| 58 | Hemodialysis | 127, 316, 144 |
| 59 | Other OR procedures on vessels of head and neck | 1, 5, 478 |
| 60 | Embolectomy and endarterectomy of lower limbs | 478, 479, 468 |
| 61 | Other OR procedures on vessels other than head and neck | 478, 479, 76 |
| 62 | Other diagnostic cardiovascular procedures | 112, 233, 7 |
| 63 | Other non-OR therapeutic cardiovascular procedures | 116, 316, 144 |
| 64 | Bone marrow transplant | 481 |
| 65 | Bone marrow biopsy | 403, 473, 395 |
| 66 | Procedures on spleen | 392, 486, 400 |
| 67 | Other therapeutic procedures; hemic and lymphatic system | 400, 401, 394 |
| 68 | Injection or ligation of esophageal varices | 201, 154 |
| 69 | Esophageal dilatation | 182, 183, 188 |
| 70 | Upper gastrointestinal endoscopy; biopsy | 174, 182, 183 |
| 71 | Gastrostomy; temporary and permanent | 14, 79, 296 |
| 72 | Colostomy; temporary and permanent | 148, 149, 468 |
| 73 | Ileostomy and other enterostomy | 148, 172, 296 |
| 74 | Gastrectomy; partial and total | 154, 155, 468 |
| 75 | Small bowel resection | 148, 149 |
| 76 | Colonoscopy and biopsy | 174, 182, 188 |
| 77 | Proctoscopy and anorectal biopsy | 188, 182, 172 |
| 78 | Colorectal resection | 148, 149, 146 |
| 79 | Local excision of large intestine lesion (not endoscopic) | 157, 158, 477 |
| 80 | Appendectomy | 167, 165, 166 |
| 81 | Hemorrhoid procedures | 157, 158, 477 |
| 82 | Endoscopic retrograde cannulation of pancreas (ERCP) | 204, 207, 208 |
| 83 | Biopsy of liver | 203, 205, 206 |
| 84 | Cholecystectomy and common duct exploration | 494, 493, 197 |
| 85 | Inguinal and femoral hernia repair | 161, 162, 163 |
| 86 | Other hernia repair | 160, 159, 154 |
| 87 | Laparoscopy (GI only) | 361, 171, 170 |
| 88 | Abdominal paracentesis | 202, 418, 205 |
| 89 | Exploratory laparotomy | 170, 171, 486 |
| 90 | Excision; lysis peritoneal adhesions | 150, 151, 365 |
| 91 | Peritoneal dialysis | 452, 316, 188 |
| 92 | Other bowel diagnostic procedures | 174, 188, 152 |
| 93 | Other non-OR upper GI therapeutic procedures | 174, 202, 188 |
| 94 | Other OR upper GI therapeutic procedures | 288, 154, 155 |
| 95 | Other non-OR lower GI therapeutic procedures | 174, 188, 189 |
| 96 | Other OR lower GI therapeutic procedures | 148, 158, 157 |
| 97 | Other gastrointestinal diagnostic procedures | 203, 170, 204 |
| 98 | Other non-OR gastrointestinal therapeutic procedures | 207, 208, 204 |
| 99 | Other OR gastrointestinal therapeutic procedures | 191, 442, 415 |
| 100 | Endoscopy and endoscopic biopsy of the urinary tract | 323, 310, 320 |
| 101 | Transurethral excision; drainage; or removal urinary obstruction | 310, 311, 477 |
| 102 | Ureteral catheterization | 323, 324, 320 |
| 103 | Nephrotomy and nephrostomy | 304, 305, 415 |
| 104 | Nephrectomy; partial or complete | 303, 305, 304 |
| 105 | Kidney transplant | 302 |
| 106 | Genitourinary incontinence procedures | 356, 309, 308 |
| 107 | Extracorporeal lithotripsy; urinary | 323, 320 |
| 108 | Indwelling catheter | 127, 320, 89 |
| 109 | Procedures on the urethra | 312, 313, 341 |
| 110 | Other diagnostic procedures of urinary tract | 331, 316, 332 |
| 111 | Other non-OR therapeutic procedures of urinary tract | 380, 373, 331 |
| 112 | Other OR therapeutic procedures of urinary tract | 303, 305, 308 |
| 113 | Transurethral resection of prostate (TURP) | 336, 337, 306 |
| 114 | Open prostatectomy | 335, 334 |
| 115 | Circumcision | 391, 390, 389 |
| 116 | Diagnostic procedures; male genital | 346, 348, 316 |
| 117 | Other non-OR therapeutic procedures; male genital | 350, 352, 418 |
| 118 | Other OR therapeutic procedures; male genital | 341, 339, 315 |
| 119 | Oophorectomy; unilateral and bilateral | 359, 358, 357 |
| 120 | Other operations on ovary | 359, 358, 384 |
| 121 | Ligation or occlusion of fallopian tubes | 374 |
| 122 | Removal of ectopic pregnancy | 378 |
| 123 | Other operations on fallopian tubes | 359, 378, 358 |
| 124 | Hysterectomy; abdominal and vaginal | 359, 358, 355 |
| 125 | Other excision of cervix and uterus | 359, 358, 360 |
| 126 | Abortion (termination of pregnancy) | 381, 383, 380 |
| 127 | Dilatation and curettage (D&C); aspiration after delivery or abortion | 381, 374, 377 |
| 128 | Diagnostic dilatation and curettage (D&C) | 364, 477, 363 |
| 129 | Repair of cystocele and rectocele; obliteration of vaginal vault | 356, 360 |
| 130 | Other diagnostic procedures; female organs | 477, 364, 363 |
| 131 | Other non-OR therapeutic procedures; female organs | 380, 384, 369 |
| 132 | Other OR therapeutic procedures; female organs | 360, 356, 378 |
| 133 | Episiotomy | 373, 372 |
| 134 | Cesarean section | 371, 370 |
| 135 | Forceps; vacuum; and breech delivery | 373, 372 |
| 136 | Artificial rupture of membranes to assist delivery | 373, 372 |
| 137 | Other procedures to assist delivery | 373, 372 |
| 138 | Diagnostic amniocentesis | 383, 379, 384 |
| 139 | Fetal monitoring | 373, 383, 379 |
| 140 | Repair of current obstetric laceration | 373, 372 |
| 141 | Other therapeutic obstetrical procedures | 384, 372, 383 |
| 142 | Partial excision bone | 231, 415, 292 |
| 143 | Bunionectomy or repair of toe deformities | 225, 477, 227 |
| 144 | Treatment; facial fracture or dislocation | 63, 185, 37 |
| 145 | Treatment; fracture or dislocation of radius and ulna | 224, 223, 252 |
| 146 | Treatment; fracture or dislocation of hip and femur | 210, 211, 249 |
| 147 | Treatment; fracture or dislocation of lower extremity (other than hip or femur) | 219, 218, 225 |
| 148 | Other fracture and dislocation procedure | 219, 218, 220 |
| 149 | Arthroscopy | 232, 415, 503 |
| 150 | Division of joint capsule; ligament or cartilage | 503, 224, 231 |
| 151 | Excision of semilunar cartilage of knee | 503, 477, 501 |
| 152 | Arthroplasty knee | 209, 471, 503 |
| 153 | Hip replacement; total and partial | 209 |
| 154 | Arthroplasty other than hip or knee | 491, 223, 228 |
| 155 | Arthrocentesis | 244, 242, 248 |
| 156 | Injections and aspirations of muscles; tendons; bursa; joints and soft tissue | 248, 277, 243 |
| 157 | Amputation of lower extremity | 113, 285, 114 |
| 158 | Spinal fusion | 498, 497, 496 |
| 159 | Other diagnostic procedures on musculoskeletal system | 216, 226, 468 |
| 160 | Other therapeutic procedures on muscles and tendons | 227, 226, 224 |
| 161 | Other OR therapeutic procedures on bone | 233, 234, 219 |
| 162 | Other OR therapeutic procedures on joints | 231, 219, 415 |
| 163 | Other non-OR therapeutic procedures on musculoskeletal system | 243, 244, 248 |
| 164 | Other OR therapeutic procedures on musculoskeletal system | 63, 213, 441 |
| 165 | Breast biopsy and other diagnostic procedures on breast | 274, 262, 259 |
| 166 | Lumpectomy; quadrantectomy of breast | 259, 260, 262 |
| 167 | Mastectomy | 258, 257, 261 |
| 168 | Incision and drainage; skin and subcutaneous tissue | 277, 278, 279 |
| 169 | Debridement of wound; infection or burn | 263, 415, 217 |
| 170 | Excision of skin lesion | 266, 269, 265 |
| 171 | Suture of skin and subcutaneous tissue | 280, 281, 141 |
| 172 | Skin graft | 217, 439, 263 |
| 173 | Other diagnostic procedures on skin and subcutaneous tissue | 283, 277, 272 |
| 174 | Other non-OR therapeutic procedures on skin and breast | 144, 316, 430 |
| 175 | Other OR therapeutic procedures on skin and breast | 268, 261, 288 |
| 176 | Other organ transplantation | 480, 103, 495 |
| 177 | Computerized axial tomography (CT) scan head | 14, 15, 141 |
| 178 | CT scan chest | 89, 88, 143 |
| 179 | CT scan abdomen | 183, 182, 204 |
| 180 | Other CT scan | 243, 236, 239 |
| 181 | Myelogram | 243, 239, 18 |
| 182 | Mammography | 276, 430, 467 |
| 183 | Routine chest X-ray | 89, 127, 143 |
| 184 | Intraoperative cholangiogram | 200, 199, 477 |
| 185 | Upper gastrointestinal X-ray | 184, 182, 183 |
| 186 | Lower gastrointestinal X-ray | 180, 184, 182 |
| 187 | Intravenous pyelogram | 323, 320, 324 |
| 188 | Cerebral arteriogram | 14, 15, 25 |
| 189 | Contrast aortogram | 130, 131, 15 |
| 190 | Contrast arteriogram of femoral and lower extremity arteries | 130, 131, 144 |
| 191 | Arterio- or venogram (not heart and head) | 78, 130, 144 |
| 192 | Diagnostic ultrasound of head and neck | 14, 15, 141 |
| 193 | Diagnostic ultrasound of heart (echocardiogram) | 127, 14, 143 |
| 194 | Diagnostic ultrasound of gastrointestinal tract | 204, 182, 208 |
| 195 | Diagnostic ultrasound of urinary tract | 316, 320, 322 |
| 196 | Diagnostic ultrasound of abdomen or retroperitoneum | 204, 182, 183 |
| 197 | Other diagnostic ultrasound | 130, 383, 277 |
| 198 | Magnetic resonance imaging | 14, 243, 15 |
| 199 | Electroencephalogram (EEG) | 26, 25, 24 |
| 200 | Nonoperative urinary system measurements | 320, 383, 89 |
| 201 | Cardiac stress tests | 143, 132, 127 |
| 202 | Electrocardiogram | 143, 127, 138 |
| 203 | Electrographic cardiac monitoring | 143, 127, 138 |
| 204 | Swan-Ganz catheterization for monitoring | 127, 144, 416 |
| 205 | Arterial blood gases | 88, 89, 127 |
| 206 | Microscopic examination (bacterial smear; culture; toxicology) | 430, 143, 390 |
| 207 | Radioisotope bone scan | 243, 239, 277 |
| 208 | Radioisotope pulmonary scan | 143, 127, 78 |
| 209 | Radioisotope scan and function studies | 143, 127, 183 |
| 210 | Other radioisotope scan | 14, 239, 243 |
| 211 | Therapeutic radiology for cancer treatment | 10, 239, 301 |
| 212 | Diagnostic physical therapy | 462 |
| 213 | Physical therapy exercises; manipulation; and other procedures | 462 |
| 214 | Traction; splints; and other wound care | 253, 254, 511 |
| 215 | Other physical therapy and rehabilitation | 462, 430 |
| 216 | Respiratory intubation and mechanical ventilation | 475, 416, 386 |
| 217 | Other respiratory therapy | 98, 88, 97 |
| 218 | Psychological and psychiatric evaluation and therapy | 430, 426, 435 |
| 219 | Alcohol and drug rehabilitation/detoxification | 435, 434, 433 |
| 220 | Ophthalmologic and otologic diagnosis and treatment | 391, 390, 389 |
| 221 | Nasogastric tube | 180, 181, 188 |
| 222 | Blood transfusion | 395, 174, 127 |
| 223 | Enteral and parenteral nutrition | 416, 79, 387 |
| 224 | Cancer chemotherapy | 410, 492, 403 |
| 225 | Conversion of cardiac rhythm | 138, 139, 127 |
| 226 | Other diagnostic radiology and related techniques | 323, 322, 320 |
| 227 | Other diagnostic procedures (interview; evaluation; consultation) | 26, 25, 141 |
| 228 | Prophylactic vaccinations and inoculations | 391, 390, 389 |
| 229 | Nonoperative removal of foreign body | 190, 189, 188 |
| 230 | Extracorporeal shock wave lithotripsy; other than urinary | 323, 204, 208 |
| 231 | Other therapeutic procedures | 391, 89, 127 |
