# Supplementary material for: What drives different treatment choices? Investigation of hospital ownership, system membership and competition
Source: Health Econ Rev. 2021 Feb 16;11:6. doi: 10.1186/s13561-021-00305-3 (PMC7885748; doi:10.1186/s13561-021-00305-3)
Supplement: Supplementary file 2 — Additional file 2: Distance Weighted Measure of Hospital Competition. [file 13561_2021_305_MOESM2_ESM.docx]

# **Appendix B**

**Distance Weighted Measure of Hospital Competition**

We use a distance-weighted method following Horwitz and Nichols [18], [44] to calculate the competition each hospital faces for each procedural group, which assigns weights by admissions and inversely by distance so that distant hospitals have less importance relative to close hospitals but may still have an effect. We measure the geodesic distance between hospitals, which uses the ellipsoidal model of earth. We also calculate the competition a hospital faces for each procedural group separately using the number of patient records of the target DRGs. Therefore, we can capture different levels of competition for different procedures. For example, a hospital might be facing high competition for cardiac related procedures but low competition for birth related procedures.

Weighted competition hospital *i* faces from hospital *k* for procedural group *l* is defined as follows:

$$C_{ikl}=\frac{n_{kl}}{\left( 1+bx_{ik}^{2} \right)^{2}}$$

where *n_kl_* is the number of patient records in the target group of procedure *l* at hospital *k*, *x_ik_* is the distance between hospital *i* and hospital *k*, and *b* is a constant. 75% of the patients of a hospital come from a disk of radius 10.4 miles and we use *b*=0.0277, which satisfies this condition following Horwitz and Nichols [18].

We allow hospitals within a state to compete with each other. We classify the hospitals as facing high and low competition from for-profit, not-for-profit, and government hospitals separately to allow for the effects of competition to differ by ownership type. Since for-profit hospitals are assumed to be expected profit maximizers and government hospitals are supposed to be market output maximizers, a hospital facing the same level of competition from profit maximizers and market output maximizers may act differently depending on its objective. Weighted competition hospital *i* faces from for-profit, not-for-profit and government hospitals for procedure *l* are denoted by $C_{il}^{F}$, $C_{il}^{N}$ and $C_{il}^{G}$ respectively and calculated as follows:

$$C_{il}^{F}=\sum_{\begin{aligned} k\in F \\ i\neq k \end{aligned}} C_{ikl}, C_{il}^{N}=\sum_{\begin{aligned} k\in N \\ i\neq k \end{aligned}} C_{ikl}, C_{il}^{G}=\sum_{\begin{aligned} k\in G \\ i\neq k \end{aligned}} C_{ikl}$$

where *F*, *N*, and *G* denote the set of for-profit, not-for-profit and government hospitals, respectively.

If the competition a hospital faces from an ownership type for a procedural group is in the top 33 percent for the ownership type and procedural group, the hospital is classified as in a high competition market for that ownership type and procedural group. If the competition a hospital faces from an ownership type for a procedural group is in the bottom 33 percent, the hospital is classified as in a low competition market for that ownership type and procedural group.
